# CXCR7/p-ERK-Signaling Is a Novel Target for Therapeutic Vasculogenesis in Patients with Coronary Artery Disease

**DOI:** 10.1371/journal.pone.0161255

**Published:** 2016-09-09

**Authors:** Zheng Cao, Xinzhu Tong, Wenhao Xia, Long Chen, Xiaoyu Zhang, Bingbo Yu, Zhen Yang, Jun Tao

**Affiliations:** 1 Department of Cardiology, Taihe Hospital, Hubei University of Medicine, Hubei, China; 2 Department of Hypertension and Vascular Disease, The First Affiliated Hospital, Sun Yat-Sen University, Guangzhou, China; Centro Cardiologico Monzino, ITALY

## Abstract

Coronary artery disease (CAD) is characterized by insufficient vasculogenic response to ischemia, which is typically accompanied by dysfunction of endothelial outgrowth cells (EOCs). CXC chemokine receptor 7 (CXCR7) is a key modulator of the neovascularization of EOCs to perfusion defect area. However, the mechanism underlying the role of EOCs in CAD-related abnormal vasculogenesis is still not clear. Here, we investigated the alteration of EOCs-related vasculogenic capacity in patients with CAD and its potential mechanism. Compared with EOCs isolated from healthy subjects, EOCs from CAD patients showed an impaired vasculogenic function *in vitro*. CXCR7 expression of EOCs from CAD patients was downregulated. Meanwhile, the phosphorylation of extracellular signal-regulated kinase (ERK), downstream of CXCR7 signaling, was also reduced. CXCR7 expression introduced by adenovirus increased the phosphorylation of ERK, which was parallel to improved function of EOCs. The enhanced adhesion and vasculogenesis of EOCs can be blocked by short interfering RNA (siRNA) against CXCR7 and ERK inhibitor PD098059. Therefore, our study demonstrates that the upregulation of CXCR7 signaling contributes to increased vasculogenic capacity of EOCs from CAD patients, indicating that CXCR7 signaling may be a novel therapeutic vasculogenic target for CAD.

## Introduction

Coronary artery disease (CAD) is the most common heart disease in China. Endothelial dysfunction has been proven to play a pivotal role in the pathogenesis of CAD [1–3]. Thus, maintaining endothelial homeostasis is important in prevention and treatment of CAD. Accumulating evidence revealed that endothelial progenitor cells (EPCs) contribute to the integrity of structure and function of endothelium [4–7]. Unfortunately, a decreased number of EPCs along with impaired function is observed in patients with CAD [8–9], which delays the progression of neovascularization response to myocardial ischemia. Although the dysfunction of EPCs in CAD patients has been reported in previous studies [10–11], the mechanism underlying this abnormal alteration remains unclear.

Endothelial outgrowth cells (EOCs), as an aging subtype of EPCs, are considered to participate in adult vasculogenesis [12–14]. EOCs appear after 4 weeks as cobblestone-shaped cells and show high proliferative potential. Recent reports focus on the possible role of EOCs in revascularization in tissue/organ suffering from ischemia [15–17] suggest that EOCs transplantation may have therapeutic approaches for ischemic cardiovascular diseases such as CAD.

Accordingly, CXC chemokine receptor seven (CXCR7) is recently identified as a novel chemokine receptor response to stromal cell-derived factor 1 (SDF-1) [18–19]. Previous study has demonstrated that CXCR7 mediates the anti-apoptosis and endothelial adhesion of renal progenitor cells, which promote renal progenitor cells to repair necrosis of kidney tissue [20]. In addition, CXCR7 also augments the adhesion of rat endothelial progenitor cells on vascular endothelium and inhibits EPC apoptosis *in vitro* [21]. These studies indicate that CXCR7 is essential for maintaining EPC function and survival. However, the molecular mechanism underlying CXCR7 mediated biological effect is still unclear. Extracellular signal-regulated kinase (ERK), a key molecular signaling in cellular proliferation and apoptosis, has been reported to contribute to the down-regulation of EPC function and pathologic progress of CAD [22]. Although it has been demonstrated that ERK functions as a downstream signaling in EPCs, the interaction between CXCR7 and ERK signaling remained further to be elucidated. Accumulating studies suggested that CXCR7 might be involved in the regulation of progenitor cell function *in vitro*.

We recently showed that CXCR7 signaling contributed to improve the reendothelialization capacity of EPCs from hypertensive patients [23]. Based on these findings, we hypothesized that diminished CXCR7 signaling might be responsible for EOCs dysfunction and ERK activation, and upregulation of CXCR7 signaling might contribute to the improvement of vascular vasculogenic capacity of EOCs in patients with CAD. Accordingly, we evaluated the alteration in CXCR7 signaling and function of EOCs in patients with CAD, and investigated the effect of upregulated CXCR7 expression on EOC-mediated vasculogenesis. The present study will enhance our understanding of the mechanisms underlying EOC dysfunction from CAD subjects, which may provide novel therapeutic targets for the prevention and treatment of CAD.

## Materials and Methods

### Subject characteristics

According to coronary angiography examination, twenty-one outpatients with newly diagnosed coronary artery disease were enrolled into the study after informed consent was obtained. Their coronary artery had stenosis (>50%) detected by quantitative coronary angiography. Patients with a history of hypertension, diabetes, peripheral artery disease, malignancy or active inflammatory disease and those with other cardiovascular risk factors were excluded. All women included were in postmenopause. Fourteen healthy subjects were enrolled into control group. The baseline characteristics of coronary artery disease and healthy subjects are shown in Table 1. All subjects were enrolled into the study after written informed consent forms were collected. This consent procedure was approved by the ethical committee of The First Affiliated Hospital, Sun Yat-Sen University (Guangzhou, China).

**Table 1:** Subjects’ Characteristics.

Groups	Control subjects (n = 14)	CAD patients (n = 21)	*P* value
Age (yrs)	58.86±7.15	61.67±4.13	0.149
Female (%)	43%	29%	0.398
BMI (kg/m2)	23.00±2.63	22.30±2.72	0.461
HR (bpm)	71.29±8.16	70.24±9.44	0.737
Systolic blood pressure (mmHg)	123.14±13.04	122.67±18.10	0.933
Diastolic blood pressure (mmHg)	75.29±10.03	73.81±12.55	0.715
Fasting plasma glucose (mmol/L)	5.44±0.64	5.09±0.45	0.086
Blood urea nitrogen (mmol/L)	5.09±1.24	5.58±1.52	0.318
Creatinine (μmol/L)	67.50±12.12	74.76±14.16	0.126
Uric acid (μmol/L)	364.46±86.96	357.86±97.26	0.843
Total cholesterol (mmol/L)	5.52±0.63	4.78±1.39	0.070
Triglyceride (mmol/L)	1.59±1.09	1.76±0.91	0.621
LDL cholesterol (mmol/L)	3.41±0.74	2.99±0.95	0.171
HDL cholesterol (mmol/L)	1.52±0.67	1.18±0.26	0.092

Data are shown as mean ± SD. CAD = coronary artery disease; HDL = high-density lipoprotein; LDL = low-density lipoprotein.

### EOCs culture and identification

Peripheral blood mononuclear cells were isolated by ficoll density gradient centrifugation as described previously [7]. EOCs were cultured and characterized by following previously described [7, 23–25]. Briefly, after 4 days culture, non-adherent cells were removed by thoroughly washing with endothelial cell basal medium-2 (EBM-2) (Clonetics). Medium was changed daily for 7 days, and then every 3 days was changed. After 4 weeks’ culture, marker proteins of cultured EPCs were examined by flow cytometry analysis using CD31 (BD) and kinase-insert domain receptor (KDR) (R&D). Based on the isolation and cultivation protocol, the adherent mononuclear cells were identified as EOCs. The method of EOC characterization was described in S1 Fig.

### CXCR7 gene transfer

After 4-weeks culture, cells were transduced with the adenovirus serotype 5 (Ad5) encoding the human CXCR7 gene (Ad5/CXCR7) or green fluorescent protein gene (Ad5/GFP) (GeneChem). After preliminary experiments were performed, human EOCs were transduced with 100 MOI Ad5/CXCR7 or Ad/GFP and incubated with EOCs medium for 48 hours before subsequent experiments for assessment of CXCR7 expression and EOCs functions.

### RNA interference

The CXCR7 siRNA (Santa Cruz) transduction was performed using Lipofectamine 2000 (Invitrogen) with following the manufacturer’s instructions. The knockdown of CXCR7 was confirmed by RT-qPCR and Western blot.

### Migration assay

EOCs migration was determined by the Wound-Healing Assay. In brief, a total of 2 × 10^5^ EOCs were plated in 6-well culture dish. A wound was created by manually scraping the cell monolayer with a p20 pipet tip, followed by wash once and supplemented with 1ml EBM-2 with or without SDF-1 (100 ng/ml, PeproTech). After 16 hours incubation at 37°C, transmigrated cells were observed under an optical microscope by independent investigators blinded to treatment groups randomly.

### Adhesion assay

Dishes were coated with fibronectin (10ug/ml). EOCs (2×10^4^) in each well of a 24-well plate were stimulated with or without SDF-1 (100ng/ml) for 5 hours at 37°C. Non-attached cells were removed with PBS, and adherent EOCs were fixed with 4% paraformaldehyde and stained with 0.3% crystal violet. The adherent EOCs were counted by independent investigators blinded to treatment groups randomly.

### Tube Formation Assay

A growth factor-reduced Matrigel (Corning) was warmed up at 4°C overnight. After completely thawed, 60μl of Matrigel was plated to 96-well plates at the same level to distribute evenly, and incubated for 1hour at 37°C. EOCs (2 x 10^4^) were resuspended with EBM-2, and loaded on the top of the Matrigel. Each conditional group contained 3 wells. Following incubation at 37°C for 2 hours, each well was imaged directly under a microscope, and an average of tubules was counted from 3–5 random fields.

### Real-time reverse transcription-quantitative PCR (RT-qPCR)

Total RNA was extracted with the High pure RNA isolation kit (TransGen). RT-qPCR was carried out by the routine 3-step method. cDNA products were amplified by the following primer pair for CXCR7 coding sequence: forward, 5’-TCTGCATCTCGACTACTCA-3’ and reverse, 5’- GTCADGCAGGACGCTTTTGTT-3’. Gene expression was analyzed by using the iQ™ SYBR Green Supermix and iQ5 Real-Time PCR detection system (Bio-Rad). The mRNA level of GAPDH gene was measured in each sample as an internal normalization standard.

### Western blot (WB)

Total EOCs protein were extracted and quantified by protein extraction reagent (Merck) and bicinchoninic acid protein assay kit (Thermo Fisher) separately. Protein extracts were subjected to SDS-PAGE, transferred to polyvinylidene fluoride membranes (Roche). The following antibodies were used: rabbit anti-CXCR7 antibody (1:250; abCAM), anti-VEGFA antibody (1:500; ImmunoWay), rabbit anti-GAPDH antibody, rabbit anti-p-ERK and anti-ERK antibody (1:1000; Cell Signaling Technology). Proteins were visualized with HRP-conjugated anti-rabbit IgG (1:2000; Cell Signaling Technology), followed by use of the ECL chemiluminescence system (Thermo).

### Statistical analysis

Results are expressed as mean value ± SD. Comparison of continuous variables in the clinical study was performed by Student’s *t* test. Comparisons between the *in vitro* experimental groups were performed using ANOVA followed by Fisher’s protected least significant difference test. In all of the analyses, *P*<0.05 was considered statistically significant. All statistical analyses used SPSS statistical software (SPSS version 20.0).

## Results

### Subject characteristics

As listed in Table 1, the baseline characteristics were not significantly different between healthy subjects and CAD patients. The characterization of endothelial outgrowth cells (EOCs) is included in S1 Fig.

### Down-regulation of CXCR7 results in reduced in vitro functions of EOCs from CAD patients

**Fig 1:**
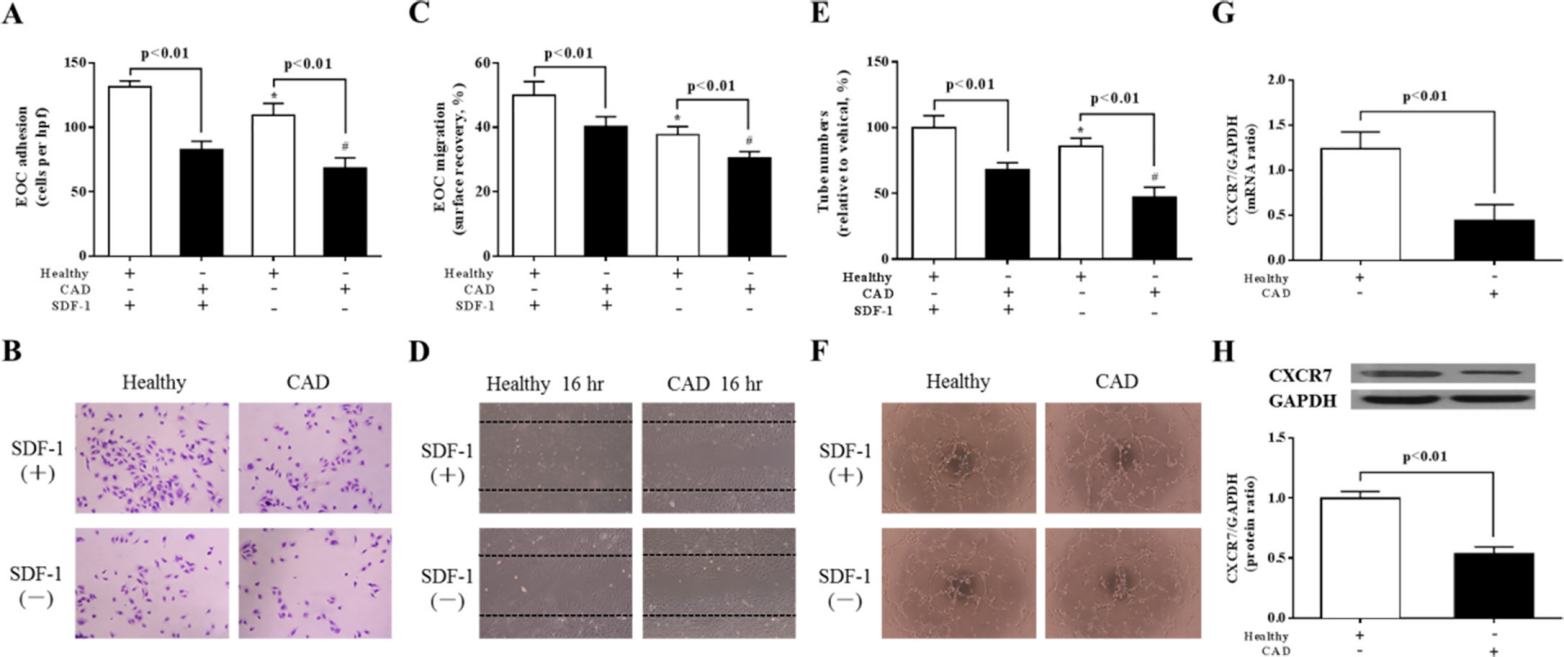
Down-regulation of CXCR7 results in reduced in vitro functions of EOCs from CAD patients. EOCs from CAD patients display reduced homing and angiogenic functions in comparison with healthy subjects. **A**-**D**: Quantification analysis (**A** and **C**) and representative photographs (**B** and **D**) of adhesion and migration of EOCs with/without SDF-1 (100 ng/ml, *P<0.01, ^**#**^P<0.01 vs. SDF-1 induced, n = 5 per group). **E**: Tube number was incorporated into tube-like structures. (*P<0.01, ^**#**^P<0.01 versus SDF-1 induced; n = 5 per group). **F**: Representative images of complete tube formation (original magnification ×10 [top] and ×10 [bottom]). **G**-**H**: Representative quantitative and photographs analyses of CXCR7 mRNA and protein expression in EOCs (n = 5 per group).

We evaluated functions of EOCs *in vitro* to investigate whether reduced expression of CXCR7 contributes to the dysfunction of EOCs in patients with CAD. Both the basal level of adhesion and migration were markedly lower in CAD-EOCs than those from healthy subjects (Fig 1A–1D). Moreover, to determine the effect on EOC vasculogenic activity, matrigel vasculogenesis assay was used to study the micro-vascular network formation of EOCs *in vitro*. We found that the tubulogenic capacity of CAD-EOCs was reduced in comparison with healthy subjects (p <0.05) (Fig 1E and 1F). Meanwhile, our data showed that the expression of CXCR7 in CAD-EOCs was significantly lower than that from healthy subjects, which was confirmed at mRNA level by RT-qPCR analysis (Fig 1G) and the protein level by western blot analysis (Fig 1H).

### CXCR7 gene transfer enhances the in vitro adhesion function and vasculogenesis capacity from CAD patients

**Fig 2:**
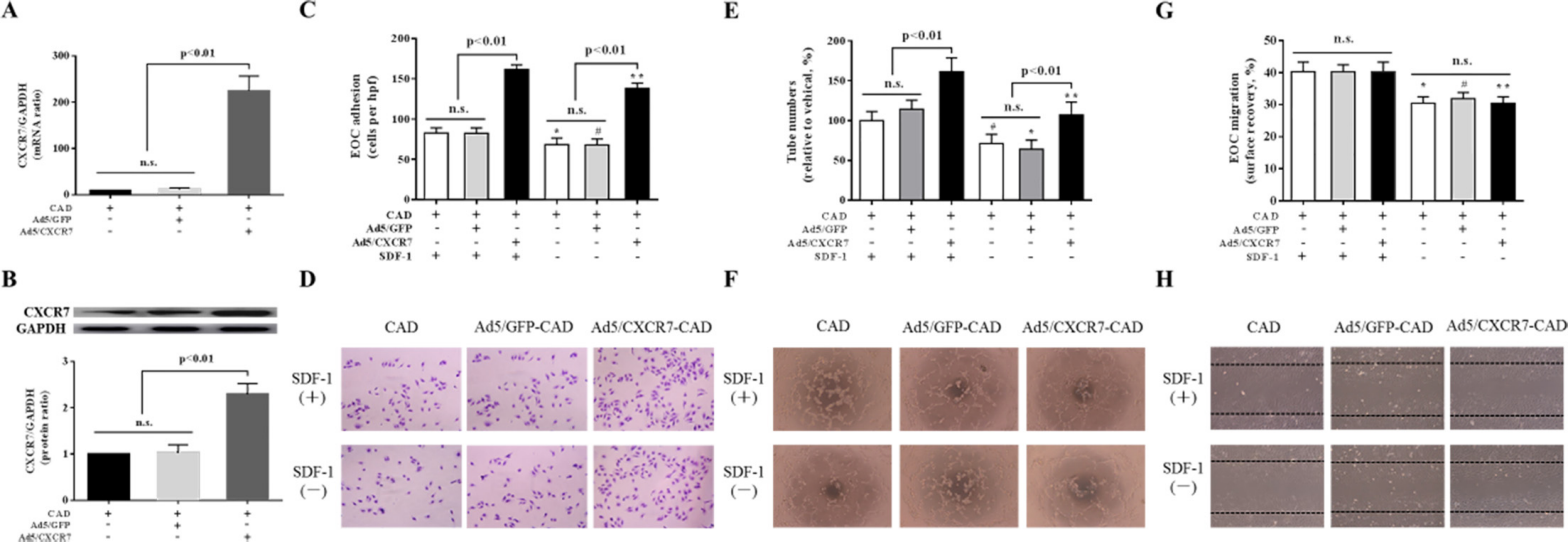
CXCR7 gene transfer enhances the in vitro adhesion function and angiogenesis capacity from CAD patients. Effects of Ad5/CXCR7 gene transfer on *in vitro* functions of CAD-EOCs. **A:** Quantitative analyses of CXCR7 mRNA level in EOCs at 48 hours after transduction measured by Real-time PCR. (n = 5 per group). **B**: Representative photographs and quantitative analyses of CXCR7 protein expression at 48 hours after transduction measured by western blot. Densitometric band analyses are quantified by Image J software and are presented as the relative ratio of CXCR7 to GAPDH. (n = 5 per group). **C**-**D**: Quantitative analyses (**C**) and representative photographs (**D**) of the adhesion activity of EOCs (*P<0.01, ^#^P<0.01, **P<0.01vs. CAD-EOCs with SDF-1; n = 5 per group). **E**-**F**: Quantification analyses (**E**) and representative photographs (**F**) of angiogenic function in the Matrigel. (*P<0.01, ^#^P<0.01, **P<0.01 vs. CAD-EOCs with SDF-1; n = 5 per group).**G**-**H**: Quantitative analyses (**G**) and representative photographs (**H**) of EOC migratory activity of EOCs (*P<0.01, ^#^P<0.01, **P<0.01 vs. CAD-EOCs with SDF-1; n = 5 per group).

In order to confirm the effect of CXCR7 on the regulation of *in vitro* functions of EOCs, we transduced CAD-EOCs with Ad5 vector encoding CXCR7 gene. After 48 hours, the CXCR7 expression was higher in Ad5/CXCR7-transduced CAD-EOCs compared with Ad5/ GFP-transduced EOCs or non-transduced EOCs from CAD patients (p <0.05) (Fig 2A and 2B), which were examined by RT-qPCR and Western blot.

In the adhesion assay, Ad5/CXCR7 gene transfer was able to improve its impaired adhesion function (Fig 2C and 2D). Moreover, Ad5/CXCR7 transfection also enhanced the vasculogenesis capacity of CAD-EOCs. As shown at Fig 2E and 2F, in comparison with CAD-EOCs and Ad5/GFP -transduced CAD-EOCs, CAD-EOCs transplanted with Ad5/CXCR7 have markedly increased tube numbers (p <0.05). However, in the migration assay, the migration capacity had no significant difference among CAD-EOCs, Ad5/GFP-transduced CAD-EOCs, and Ad5/CXCR7-transduced CAD-EOCs (p <0.05) (Fig 2G and 2H). SDF-1 induced can significantly enhance adhesion, migration and vasculogenesis of EOCs (Fig 2C–2H).

### CXCR7/ERK signaling influences vasculogenesis and VEGFA expression of EOCs

**Fig 3:**
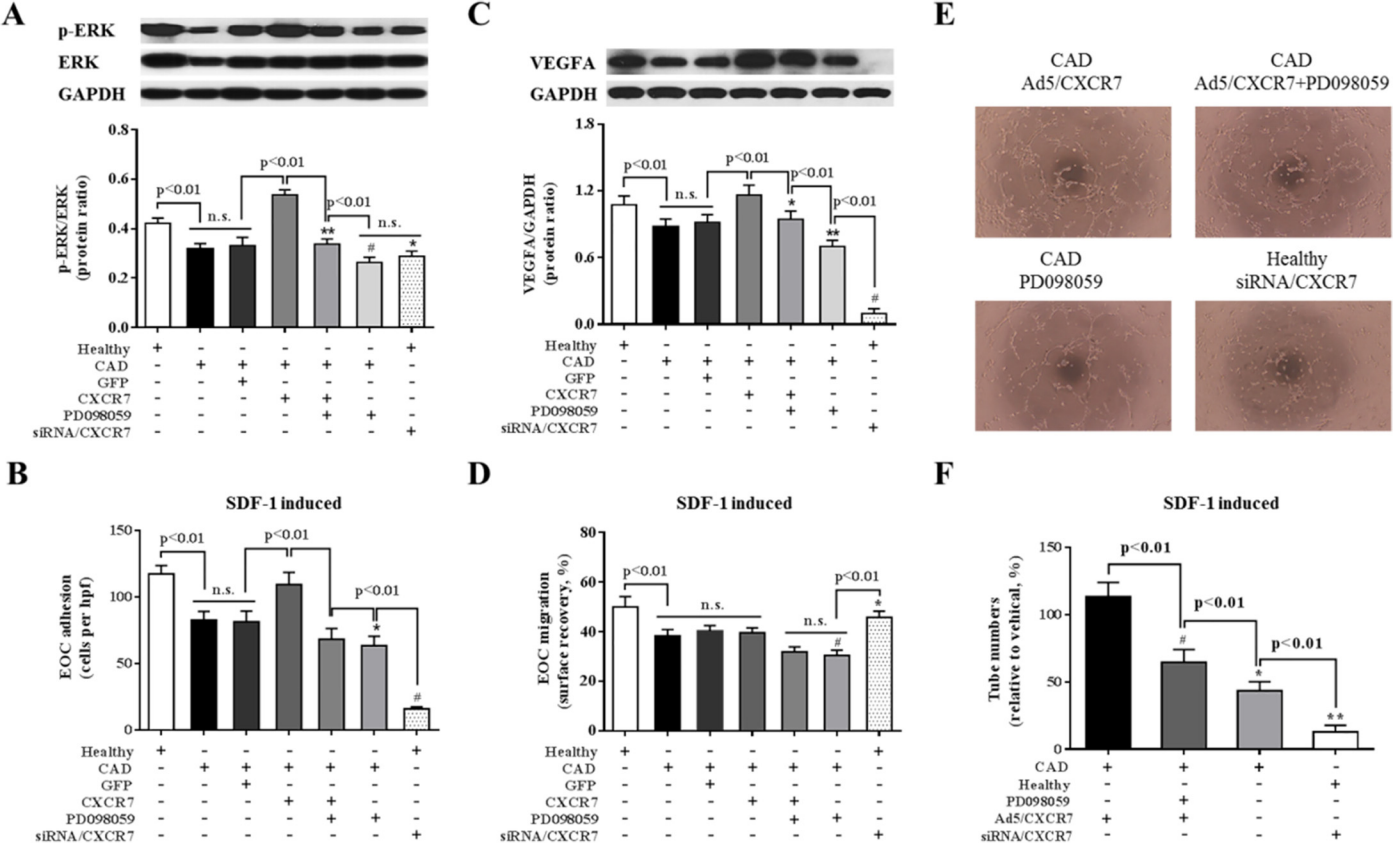
CXCR7/ERK signaling influences angiogenesis and VEGFA expression of EOCs. CXCR7/p-ERK signaling is involved in the reduced functions of CAD-EOCs. **A**: Representative photographs and quantitative analyses of p-ERK protein expression in EOCs at 48 hours after treatments (**P<0.01, ^#^P<0.01 vs. Ad5/CXCR7-CAD EOCs; *P<0.01 vs. healthy EOCs and Ad5/CXCR7-CAD EOCs; n = 5 per group). **B**: Representatively quantitative analyses of the adhesion activity of EOCs with SDF-1 (*P<0.01 vs. CAD-EOCs; ^#^P<0.01vs. healthy EOCs or Ad5/CXCR7-CAD EOCs; n = 5 per group). **C**: Representative photographs and quantitative analyses of VEGFA protein expression in EOCs at 48 hours after treatments (*P>0.01 vs. CAD-EOCs;**P<0.01 vs. Ad5/CXCR7-CAD EOCs and CAD-EOCs; ^#^P<0.01 vs. healthy EOCs and Ad5/CXCR7-CAD; n = 5 per group). **D**: Representatively quantitative analyses of the migratory activity of EOCs with SDF-1 (^#^P<0.01vs. CAD-EOCs; *P>0.01 vs. healthy EOCs; n = 5 per group). **E**-**F**: Representative photographs (**E**) and quantification analyses (**F**) of angiogenic function in the Matrigel. (^#^P<0.01, *P<0.01, **P<0.01 vs. Ad/CXCR7-CAD EOCs; n = 5 per group).

To investigate the effect of CXCR7/ERK signaling on EOC vasculogenesis, we tested tube formation and VEGFA level of EOCs. Consistent with CXCR7, the expression of p-ERK and VEGFA was significantly lower in CAD-EOCs compared with healthy subjects (*P*<0.01). Moreover, our data indicated that increased CXCR7 expression of CAD-EOCs by Ad5/CXCR7 gene transfer could enhance the expression of p-ERK and VEGFA. PD098059, an inhibitor of ERK, was capable to lower VEGFA level upregulated by Ad5/CXCR7 gene transfer, and siRNA/CXCR7 could down-regulate expression of VEGFA of EOCs from healthy subjects (Fig 3A and 3C). Meanwhile, PD098059 could also attenuate adhesion and vasculogenesis of EOCs *in vitro* with CXCR7 overexpression and stimulated with SDF-1, and siRNA/CXCR7 would reduce the activity of healthy EOCs (Fig 3B, 3E and 3F), but migration of EOCs did not (Fig 3D).

## Discussion

Our present study demonstrated that expression of CXCR7 in EOCs from CAD patients was significantly lower than that from healthy subjects, which could be lead to the impaired *in vitro* adhesion, vasculogenic capacity of EOCs from CAD patients. Upregulation of CXCR7 expression significantly restored the functions of EOCs from CAD patients, augmented expression of VEGFA and ultimately leaded to improved EOC-mediated *in vitro* vasculogenesis. The improved function of EOCs was inhibited by siRNA/CXCR7 or ERK inhibitor PD098059, and ERK modulated EOC function by declining the phosphorylation of ERK. For the first time, our study demonstrated that diminished CXCR7 expression with downregulation of p-ERK/VEGFA signaling pathway, which was, at least in part, involved in impaired function of EOCs from CAD patients. CXCR7/p-ERK signaling played a key role in the deterioration of vasculogenic capacity in presence of CAD and upregulation of CXCR7 can at least in part restored the unfavorable alteration.

It is well recognized that EPCs from CAD patients has an impaired function in comparison with healthy subjects [26–27]. Consistent with prior studies [23, 28–29], our data showed that both EOC migration toward SDF-1 and adhesion were markedly impaired in CAD patients. Moreover, EOCs from the CAD patients exhibited a significantly reduced vasculogenic capacity in the injured carotid artery than those from the healthy subjects, suggesting a reduction in endogenous neovascularization ability in CAD patients derived EOCs. Recently, increased attention is paid to the role of CXCR7 in modulating EPC function. It has been reported that CXCR7 is essential for maintaining the *in vitro* adhesion, proliferation and vasculogenic ability of EPCs [28]. Indeed, our study revealed that CXCR7 contributed to the dysfunction of EOCs from CAD patients. Interestingly, Ad5/CXCR7 gene transfer induced elevation of CXCR7 expression, and improved the impaired *in vitro* adhesion and vasculogenic activity of EOCs. This effect can be significantly blocked by siRNA/CXCR7. These findings further demonstrate an important role of CXCR7 in maintaining the function of EOCs from CAD patients. However, the exact molecular mechanism responsible for the CXCR7-enhanced upregulationn of vasculogenic capacity remains to be further studied.

Notably, ERK, a widely expressed signaling molecular, is pivotal for cellular proliferation, differentiation and survival in various cells, including EPCs [30–32], which can be activated during cell angiogensis induced by Toll-like receptor 2 in endothelial cells [33]. Our data demonstrated that phosphorylated ERK expression in EOCs from CAD patients was significantly lower compared with healthy subjects, and upregulation of CXCR7 by gene transfer can reverse the decreased phosphorylated ERK level in EOCs from CAD patients, indicating that ERK was the downstream signaling of CXCR7 pathway in EOCs. Furthermore, when the ERK signaling of EOCs was blocked by ERK antagonist, the increased EOCs function mediated by upregulation of CXCR7 was inhibited. These results revealed that ERK signaling pathway was involved in the CXCR7-mediated regulation of EOCs function.

Additionally, our results showed that paralleled to CXCR7 expression, VEGFA level of EOCs was significantly lower in EOCs from CAD patients compared with healthy EOCs. Ad5/CXCR7 gene transfection augmented the expression of VEGFA in EOCs from CAD patients, and the effect can be inhibited by blockage of CXCR7/ERK signaling pathway. It is well known that VEGF plays an important role in migration, proliferation and vasculogenic capacity of EPCs. Thus, we inferred that VEGFA may be the ultimate signal of CXCR7/ERK pathway, which exerts the beneficial effects on EOCs function.

The findings presented in this study have important clinical implications. Impaired EPC function is not just the initial step, but also one of the major factors leading to the poor prognosis of CAD [34, 35]. Our study showed that the restored capacity of EOCs from CAD patients was impaired and upregulation of CXCR7 in these EOCs can restore the *in vitro* adhesive and vasculogenic abilities of EOCs. EOC adhesion to the extracellular matrix is one of the key steps in the process of neovascularization [36]. The augmentation of adhesive ability in EOCs from CAD patients can increase the chance of EOCs “planting” in the injury sites and accelerate endothelial regeneration. Based on the current data taken with previous studies, CXCR7 may be an important target to increase vasculogenic capacity of EOCs for therapeutic approaches of CAD.

There are some limitations in this present study. First, at least two populations of EPCs have been reported, that is, early EPCs and late EPCs (EOCs) obtained after several weeks of culture. It has been showed that late EPCs are more important for the repair of vasculogenesis [37]. Therefore, our study only focused on the role of CXCR7/p-ERK signaling pathway in the modulation of EOCs function. The alteration in CXCR7/p-ERK signaling pathway in the early EPCs remains to be elucidated. Second, CXCR4 is the other major regulation factor in the EPCs as SDF-1 receptor and our data showed that CXCR7/p-ERK signaling pathway exerted a significant impact on vasculogenesis activity of EOCs. Given the common role of these pathways in EPC regulation, further study is needed to understand the crosstalk between CXCR4 and CXCR7. Third, we only pay attention to the CXCR7/p-ERK signaling pathway in EOCs related to vasculogenesis in vitro. Therefore, in vivo research involved in vasculogenesis capacity of EOCs need to be further study.

In summary, our present study demonstrates that for the first time CXCR7/ERK signaling pathway is related to impaired EOC function in CAD patient. Upregulation of CXCR7 by gene transfer can restore EOC function and ultimately enhance *in vitro* neovascularization. These findings suggest that CXCR7 is an important regulation target of vasculogenesis in CAD, providing a novel cell-based therapeutic strategy for cardiovascular disease.

## Supporting Information

S1 Fig The characterization of cultured EOCs from CAD patients. Flow cytometry analysis (FACS) of the endothelial markers for CD31 and KDR. Double FACS against CD31 and KDR after 4 weeks of culture (E), CD31 cells (A and C), and KDR cells (C and D) in the same condition (n = 3, p<0.05).
(TIF)

## Abbreviations

CAD: coronary artery disease
EPCs: endothelial progenitor cells
EOCs: endothelial outgrowth cells
SDF-1: stromal cell-derived factor-1
CXCR7: CXC chemokine receptor seven
ERK: extracellular signal-regulated kinase
p-ERK: phosphorylated extracellular signal-regulated kinase
Ad5: adenovirus serotype 5
GFP: green fluorescent protein
