# Supplementary figures and images for: CXCR7/p-ERK-Signaling Is a Novel Target for Therapeutic Vasculogenesis in Patients with Coronary Artery Disease
Source: PLoS One. 2016 Sep 9;11(9):e0161255. doi: 10.1371/journal.pone.0161255 (PMC5017667; doi:10.1371/journal.pone.0161255)

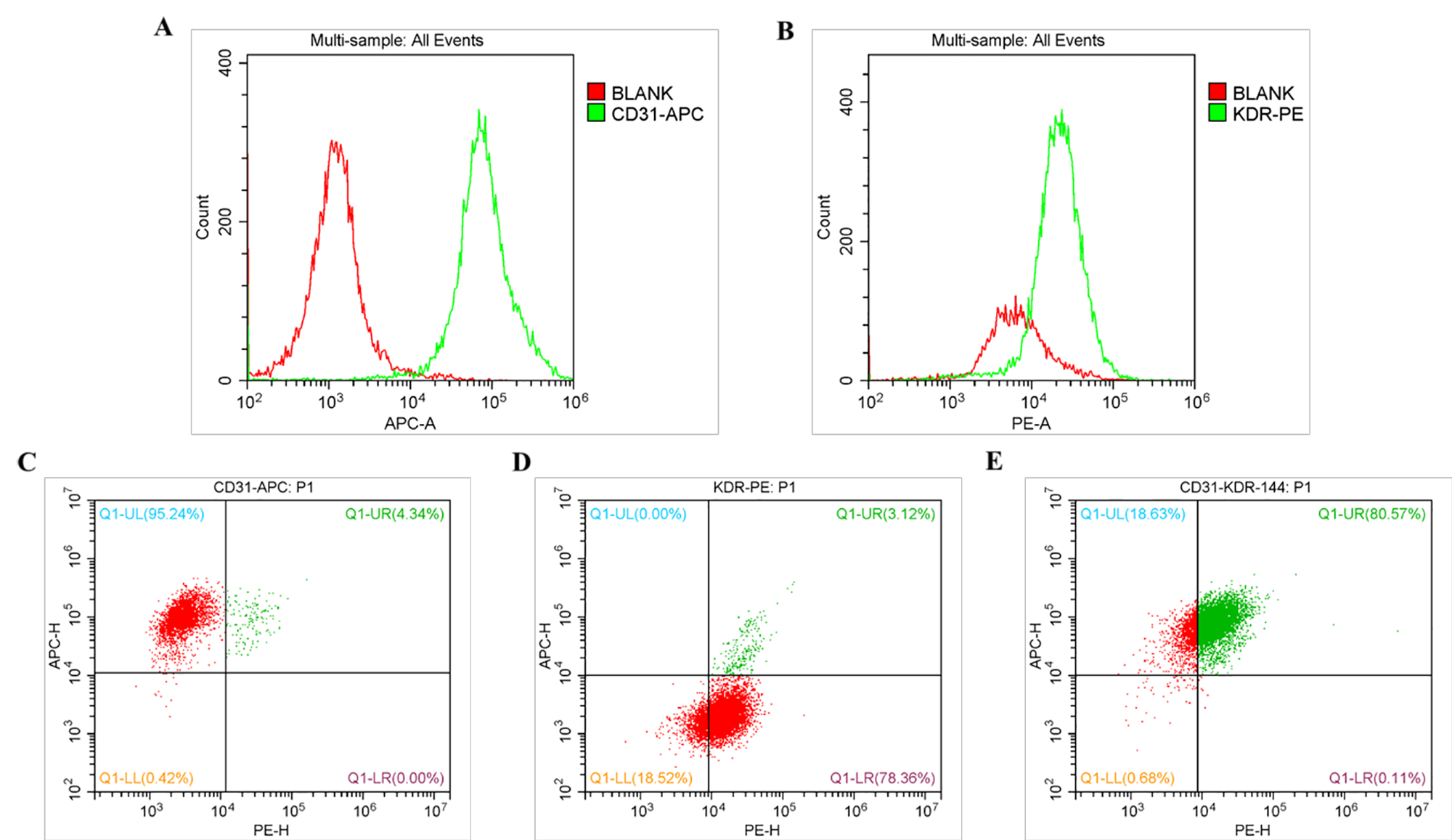

Supplement: S1 Fig — Flow cytometry analysis (FACS) of the endothelial markers for CD31 and KDR. Double FACS against CD31 and KDR after 4 weeks of culture (E), CD31 cells (A and C), and KDR cells (C and D) in the same condition (n = 3, p<0.05). (TIF) [file pone.0161255.s001.tif]
